# A lesson from the wild: The natural state of eosinophils is Ly6G^hi^

**DOI:** 10.1111/imm.13413

**Published:** 2021-09-15

**Authors:** Iris Mair, Andrew Wolfenden, Ann E. Lowe, Alex Bennett, Andrew Muir, Hannah Smith, Jonathan Fenn, Janette E. Bradley, Kathryn J. Else

**Affiliations:** ^1^ Lydia Becker Institute of Immunology and Inflammation School of Biological Sciences Faculty of Biology, Medicine and Health University of Manchester Manchester UK; ^2^ School of Life Sciences University of Nottingham Nottingham UK

**Keywords:** ecoimmunology, eosinophils, flow cytometry, granulocytes, Ly6G, phenotype, wild mice

## Abstract

With a long history of promoting pathological inflammation, eosinophils are now emerging as important regulatory cells. Yet, findings from controlled laboratory experiments so far lack translation to animals, including humans, in their natural environment. In order to appreciate the breadth of eosinophil phenotype under non‐laboratory, uncontrolled conditions, we exploit a free‐living population of the model organism *Mus musculus domesticus*. Eosinophils were present at significantly higher proportions in the spleen and bone marrow of wild mice compared with laboratory mice. Strikingly, the majority of eosinophils of wild mice exhibited a unique Ly6G^hi^ phenotype seldom described in laboratory literature. Ly6G expression correlated with activation status in spleen and bone marrow, but not peritoneal exudate cells, and is therefore likely not an activation marker *per se*. Intermediate Ly6G expression was transiently induced in a small proportion of eosinophils from C57BL/6 laboratory mice during acute infection with the whipworm *Trichuris muris*, but not during low‐dose chronic infection, which better represents parasite exposure in the wild. We conclude that the natural state of the eosinophil is not adequately reflected in the standard laboratory mouse, which compromises our attempts to dissect their functional relevance. Our findings emphasize the importance of studying the immune system in its natural context – alongside more mechanistic laboratory experiments – in order to capture the entirety of immune phenotypes and functions.

## Graphical abstract

Laboratory studies have ascribed eosinophils and eosinophil subsets important roles in tissue development, homeostasis and repair, yet their phenotype and function remain unclear in animals under natural conditions. Wild mice harbour a large pool of Ly6G^hi^ eosinophils, a phenotype rarely reported in laboratory studies and only partially explained by activation status or parasitic infections. Studying house mice in their natural environment can offer insights into environmental factors that have shaped the immune system across evolutionary timescales, complementing more traditional, mechanistic laboratory studies.

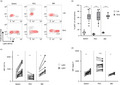

**Abbreviations**
BM: Bone marrow
FACS: Fluorescence‐activated cell sorting
MFI: Mean fluorescent intensity
PEC: Peritoneal exudate cells
SPF: Specific pathogen‐free

## INTRODUCTION

Eosinophils have long been considered destructive end‐stage effector cells. They promote allergic inflammation, and whilst helminth infections in humans are typically associated with eosinophilia, their role in the anti‐helminth responses is controversial [1]. Eosinophil‐deficient mouse models have been studied for over 20 years and have largely failed to show any overt signs of ill health [2]. The absence of adverse effects unambiguously caused by depletion of eosinophils following the growing trend of anti‐IL‐5 treatment in humans suffering from severe asthma paves the way for the conclusion that this immune cell type is redundant in humans [2]. Nevertheless, eosinophils or eosinophil‐like cells are conserved across all vertebrate species, meaning that over the past 450 million years since the diversification of vertebrates, no vertebrate species has lost this cell type along its evolutionary path. Yet, an important concept in ecological theory is the trade‐off between immune investment and fitness, termed ‘immune trade‐off’, that is that immune responses are energetically costly and that a higher investment in the immune system can lead to a lower reproductive success [3]. It is therefore highly likely that the eosinophil plays as yet unappreciated roles under naturally occurring circumstances, to justify its ubiquitous presence in the phylum Chordata.

The prevailing view of the eosinophil as a purely proinflammatory cell type has been challenged in recent years with a growing number of laboratory mouse studies describing non‐redundant roles in tissue development, homeostasis and repair. This includes a variety of tissues and cell types, for example maintenance of mucosal homeostasis [4], contractility of blood vessels [5], control of glucose metabolism in adipose tissue [6], liver regeneration [7], and wound healing of injured muscle tissue [8] including following myocardial infarction [9]. However, whilst advances have been made in understanding eosinophil contribution to health in laboratory animals, eosinophil functions in humans are largely undefined [10]. Thus, the equivalent roles seen in laboratory mice have not established in man despite processes such as tissue homeostasis and repair being likely more relevant, as well as complex, in a free‐living animal (including humans) than in a laboratory‐kept animal, due to the range of environmental and infectious challenges with which they must contend.

The laboratory mouse, the model system mainly used for immunologically research, is kept under specific pathogen‐free (SPF) conditions, with no immune or other challenges in life apart from the researcher's experimental design. The peripheral immune system of laboratory mice therefore more closely resembles that of a human newborn than that of an adult [11]. There have been several approaches to recreate a more variable context in laboratory mice by introducing factors encountered by animals in the wild, for example the exposure to a more natural environment [12, 13], sequential infections [14], co‐housing of naïve laboratory mice with ‘dirty’ pet mice [11] or direct transfer of wild microbiota into SPF mice [15]. These incremental environmental changes led to the immune system of the laboratory mouse gaining maturity [12, 13, 14], resembling more that of an adult human [11] and bestowing a greater resistance to a variety of experimental disease challenges [11, 14, 15]. Critically, the immune system of laboratory mice matured by the presence of a wild mouse microbiome modelled more closely the immune responses observed in human volunteers in failed clinical trials than the same studies carried out in conventional laboratory mice [16]. Whilst the inclusion of several abiotic and biotic factors into a laboratory mouse experiment are possible – and embraced successfully in barn and outdoor enclosure settings [17] – the natural environment of an animal including diet, infections, social interactions, reproductive cycles and seasonal changes is impossible to emulate in a laboratory setting. In particular, highly context‐dependent processes such as homeostasis and tissue repair are likely influenced by a combination of factors. It is noteworthy that studying the immune system in wild animals comes with great logistical challenges that make these studies more akin to long‐term observational ecological studies. Furthermore, there is limited scope for interventions and mechanistic studies due to animal welfare concerns in a natural setting where released animals cannot be monitored closely and may not be re‐trapped, as well as concerns about disrupting the ecological balance. Studies of immunology in wild animals should therefore be seen as complementary to traditional laboratory‐based studies, which enable mechanistic insight but which themselves have limitations in that they cannot fully capture environmental variation. Thus, we strongly believe that investigations aiming to define eosinophil function will benefit from an incorporation of the environmental factors that shaped the immune system of vertebrates across evolutionary timescales. We here present to our knowledge for the first time the phenotypic data on the eosinophil in a wild rodent, a proof of principle of the feasibility of in‐depth immunological analysis of a free‐living model system and of the potential that this study system has to offer.

## MATERIALS AND METHODS

### Mice

Wild house mice were live‐trapped between September and December 2019 on the Isle of May (56°11′11·6″N, 2°33′24·1″W) on two trapping grids of 96 Longworth traps, each placed 8–10 metres apart in a 6 × 16 grid and containing Sizzle Nest (Datesand, catalogue number CS1A09) and sunflower seeds (Figure S3). Male C57BL/6 mice were bought from Envigo or Charles River and maintained at a temperature of 20–22°C in a 12‐h light–12‐h dark lighting schedule, in sterile, individually ventilated cages in same‐sex groups of 2–5, with food and water *ad lib*. Laboratory mice were 8–13 weeks old when used for this study. All animals used for this study were euthanized by a rising concentration of CO_2_.

### Infection of laboratory mice with *Trichuris muris*

Mice were infected with *T*. *muris* eggs via oral gavage in a final volume of 200 μl of deionized water. For low‐dose *T*. *muris* infection, 30 embryonated eggs were given to each mouse, and for high‐dose infection, 100 infective embryonated eggs were given. Parasite maintenance, assessing of egg infectivity and counting of eggs were performed as described previously [18].

### Tissue preparation and cell isolation

Peritoneal exudate cells (PECs) were obtained by washing of the peritoneal cavity with 5 ml sterile PBS. Femurs were sterilized in 70% ethanol for 1 min prior to flushing of bone marrow (BM) using sterile PBS. Spleens were manually dissociated through 70‐μm filters and red blood cells lysed. Cells were counted using haemocytometers and 0·4% nigrosin (Sigma‐Aldrich) dilutions for the exclusion of dead cells on the Isle of May, and using a CASY cell counter (Scharfe System) at the University of Manchester.

### Flow cytometry

Single‐cell suspensions were stained with Fixable Viability Dye eFluor 455UV and anti‐CD16/CD32 (both from Thermo Fisher) in PBS prior to addition of the relevant fluorochrome‐conjugated antibodies in FACS buffer supplemented with Super Bright staining buffer (Thermo Fisher). The following antibodies were used: Life Technologies: CD11c‐SB436 (clone N418), F4/80‐eFluor 506 (clone AG BM8), Sca‐1‐SB600 (clone D7), CD11b‐SB780 (clone M1/70), CD86‐FITC (clone GL1), Ly6C‐PerCP‐Cy5.5 (clone HK1.4), CD68‐PE (clone FA‐11), MHC‐II‐PE‐eFluor 610 (clone IA/IE), CD206‐PE‐Cy7 (clone (MR6F3), CD64‐APC (clone AFS98), CD45‐Af700 (clone 104), CD3‐APC‐ef780 (clone 145‐2C1), NKp46‐APC‐ef780 (clone 29A1.4), CD19‐APC‐ef780 (clone HIB19; BD), Siglec‐F‐SB645 (clone E50‐2440) and Ly6G‐SB702 (clone 1A8). Samples were acquired on an LSRFortessa running FACSDiva 8 software (Becton Dickinson, Wokingham, UK). Data were analysed using FlowJo software (TreeStar; version 10.4.2) (Figure S4). For samples with fewer than 1,000 total eosinophils acquired, data were not used to compare Ly6G^+^ and Ly6G^−^ subsets.

### ImageStream

For ImageStream analysis, the following antibodies were used: Thermo Fisher; Fixable Viability Dye eFluor‐780 (catalogue number 65‐0865‐14), anti‐CD16/CD32 (clone 93), CD3‐APC‐ef780 (clone 145‐2C1), NKp46‐APC‐ef780 (clone 29A1.4), CD19‐APC‐ef780 (clone HIB19), F4/80‐eFluor 506 (clone AG BM8), CD11b‐PE (clone M1/70; BD), Siglec‐F‐SB645 (clone E50‐2440; Biolegend) and Ly6G‐PerCPCy5.5 (clone 1A8). Data acquisition was performed on ImageStream X (Amnis/EMD Millipore, Seattle, WA) running INSPIRE (version 200.1.681.0). Images of cells were acquired with a 60× objective. Eosinophils were identified as Siglec‐F^+^F4/80^int^ and macrophages as Siglec‐F^−^F4/80^hi^ events after removal of lineage‐positive events (CD3, CD19, NKp46), dead cells, and neutrophils (Ly6G^hi^ Siglec‐F^−^ F4/80^−^). All data analysis was performed using the IDEAS^®^ software version 6.

### Cell sorting

Single‐cell suspensions were stained as described above for ImageStream analysis. Cells were sorted on a BD Influx (Becton Dickinson, Wokingham, UK) for Siglec‐F^+^ F4/80^int^ cells, and isolated cells were collected in PBS and stored at 4°C prior to performing cytospin. Data acquisition, compensation and sorting were all performed in Sortware software v3 (BD Biosciences).

### Immunofluorescence

FACS‐sorted Siglec‐F^+^ F480^int^ cells were cytospun, unspecific binding was blocked for 30 min with PBS and 1% BSA (Sigma), and then, biotinylated anti‐Siglec‐F antibody (R&D, clone BAF1706) was added for 60 min at room temperature. Washing steps, addition of Streptavidin‐HRP solution (R&D) and Tyramide‐AF594 (Invitrogen) were performed according to the manufacturer's instructions. Slides were mounted using ProLong Antifade reagent/Vectashield, containing DAPI (Molecular Probes). Slides were imaged on a Nikon Eclipse Ci Microscope using a 40× Plan Fluor objective, captured using a Nikon DS‐Fi3 camera and NIS‐Elements software (Nikon Metrology UK Ltd., Derby, UK) and visualized using *Fiji ImageJ* (http://imagej.net/Fiji/Downloads).

### Data presentation and statistical analyses

Paired data are indicated by a connecting line between two data points. Comparisons between groups were undertaken using Prism (7.0; GraphPad Software). Statistical tests used are denoted in figure legends. Significance was set at *p ≤ 0·05, **p ≤ 0·01 and *** *p* ≤ 0·001.

## RESULTS AND DISCUSSION

**FIGURE 1:**
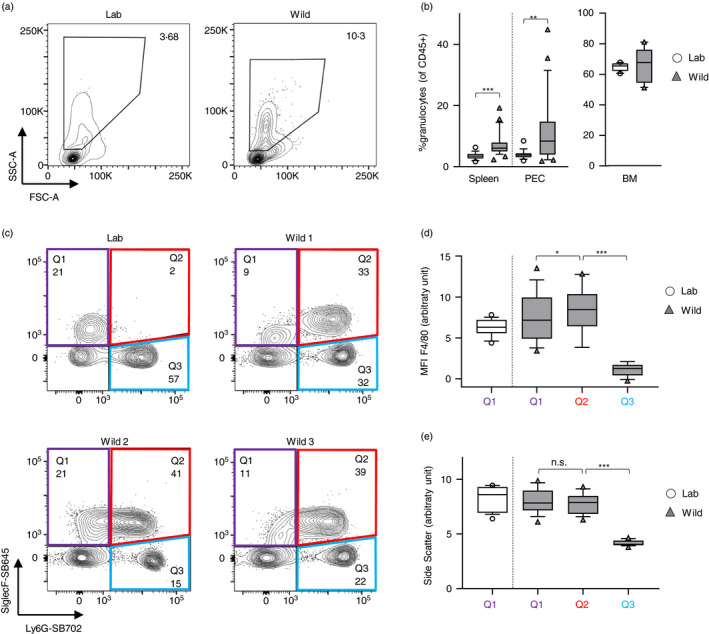
Enriched granulocyte population in wild mice contains a prominent Siglec‐F^+^Ly6G^+^ cell population. Spleens, peritoneal exudate cells (PEC) and bone marrow (BM) were collected from wild house mice from the Isle of May between September and December 2019, as well as from naïve C57BL/6 mice. (a) Representative flow cytometric plots of SSC^hi^ granulocyte population within CD45^+^ cells in laboratory and wild mouse spleens. (b) Proportion of granulocytes in laboratory and wild mice. (c) Representative flow cytometric plots showing Siglec‐F and Ly6G expression within splenic granulocyte population. (d) Mean fluorescent intensity (MFI) of F4/80 expression levels and (e) mean side‐scatter characteristic of the granulocyte subpopulations as defined in (c). Box plots show median and quartiles with 10–90 percentile and residuals as dots. Laboratory versus wild data were analysed using a two‐tailed Mann–Whitney *U*‐test; matched F4/80 and side‐scatter data were analysed using a Friedman test with Dunn's multiple comparison test. n(Lab) = 16 (Spleen, PEC), 10 (BM); n(Wild) = 25 (Spleen, PEC), 10 (BM)

To make a first step towards understanding eosinophil distribution, function and phenotype in the wild, we studied a free‐living population of house mice on a Scottish island [19] and compared our findings with naïve laboratory mice. To date, the majority of wild rodent studies explore adaptive immune responses with few publications focussing on myeloid cell types [20]. Granulocytes have been shown to be elevated in the blood or lymphoid organs of free‐living or non‐pathogen‐free housed mice [11, 12, 13], but eosinophil proportion or phenotype was not reported. We compared three distinct tissues, namely spleen, peritoneal exudate cells (PEC) and bone marrow (BM) of wild and laboratory mice and found an enriched pool of granulocytes in spleen and PEC, but not BM of wild mice (Figure 1a,b). As expected, laboratory splenic granulocytes consisted mainly of Siglec‐F^−^Ly6G^+^ neutrophils with around 20% Siglec‐F^+^Ly6G^−^ eosinophils (Figure 1c). However, to our surprise, the majority of wild splenic granulocytes presented with a Siglec‐F^+^Ly6G^+^ double‐positive phenotype, which either appeared as a continuum from the Siglec‐F^+^Ly6G^−^ eosinophil population or could be seen as a population distinct from Ly6G^−^ eosinophils (Figure 1c). In this context, Abolins et al. have previously described the presence of a putative novel myeloid cell type in the spleen of wild mice [13]. In this paper, CD11b^+^CD11c^−^ myeloid cells were teased apart by their F4/80 and Ly6G expression and revealed a population of F4/80^+^Ly6G^mid−hi^, which was only seen in wild mice, and, based on the cells' high average side scatter, was named ‘hyper‐granulocytic myeloid cell’ (HGMC). The surface marker Siglec‐F, commonly used to identify eosinophils in laboratory mice, was not included in Abolins' study, and eosinophils were therefore not accounted for. Based on a comparable F4/80, Ly6G and side‐scatter profile of the cell population described here (Figure S1), we concluded our cell population was the same as that reported in Abolins et al. [13].

**FIGURE 2:**
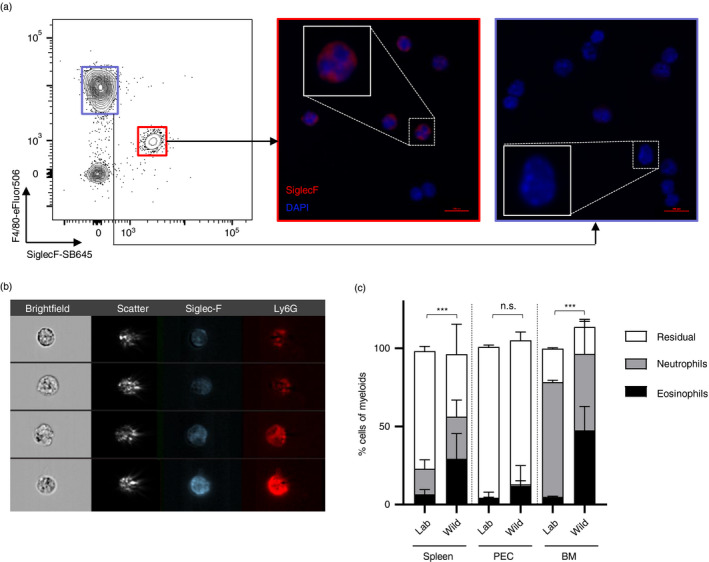
Wild house mice harbour a large pool of eosinophils in spleen and bone marrow. Spleens, peritoneal exudate cells (PEC) and bone marrow (BM) were collected from wild house mice from the Isle of May between September and December 2019, as well as from naïve C57BL/6 mice. (a) PEC cells from wild mice FACS sorted on either Siglec‐F^+^F4/80^int^ or Siglec‐F^−^F4/80^hi^ were cytospun and stained with DAPI (blue) and anti‐Siglec‐F antibody (red); *n* = 1. (b) ImageStream analysis of wild mouse splenic Siglec‐F^+^ cells, representative of 5 mice. (c) Proportion of Siglec‐F^+^F4/80^int^ eosinophils and Siglec‐F^−^F4/80^−^Ly6G^+^ neutrophils among the myeloid cell population in laboratory and wild mice. Box plots show median with interquartile range. Laboratory versus wild eosinophil proportions were analysed using a two‐tailed Mann–Whitney *U*‐test; n(Lab) = 16 (Spleen, PEC), 10 (BM); n(Wild) = 25 (Spleen, PEC), 10 (BM)

In order to classify this novel population as either eosinophils, neutrophils or a novel cell type altogether as suggested by Abolins et al., we compared the phenotypes of Siglec‐F single‐positive, Ly6G single‐positive and Siglec‐F/Ly6G double‐positive myeloid cells. The marker F4/80, typically differentially expressed by eosinophils and neutrophils, as well as side scatter of the double‐positive population, showed a closer resemblance with the Siglec‐F single‐positive population and was significantly different to SSClo, F4/80‐ neutrophils (Figure 1d,e). We therefore hypothesized that these double‐positive cells were eosinophils with an unappreciated phenotype, rather than a neutrophil subset or a novel cell type. We analysed the total Siglec‐F^+^ population in wild mice further to ascertain their correct classification, as other immune cell types including alveolar macrophages [21] and mast cells [22] have been reported to be able to express Siglec‐F. In order to visualize the nucleus of these cells, splenocytes stained and fixed in the field were sorted by fluorescence‐activated cell sorting (FACS), cytospun and stained for DAPI and Siglec‐F^+^ by immunofluorescence. Siglec‐F^+^ cells presented with a multi‐lobate nucleus (Figure 2a), consistent with their phenotypic classification as eosinophils and excluding the possibility that they were mast cells or any non‐polymorphonuclear leucocyte. ImageStream analysis further confirmed brightfield and scatter characteristics of Siglec‐F^+^ cells that are typical for eosinophils, even when expressing Ly6G (Figure 2b). Although cell sorting of live cells was not feasible due to logistical limitations of the fieldwork, precluding more detailed population analyses such as transcriptional profiling or functional assays of the Ly6G^−^ and Ly6G^+^ eosinophil populations, collectively our cellular phenotyping describes a unique Ly6G^hi^ phenotype of wild mouse eosinophils. As summarized in Table S1, CD11c was also differentially expressed on wild compared with laboratory mouse eosinophils, albeit not as markedly as Ly6G (data not shown). Notably, eosinophils – Ly6G^−^ and Ly6G^+^ combined – were over fourfold more prevalent within myeloid cells of the spleens and over 10‐fold more prevalent in the myeloid compartment of the bone marrow of wild mice compared with laboratory mice (Figure 2c).

**FIGURE 3:**
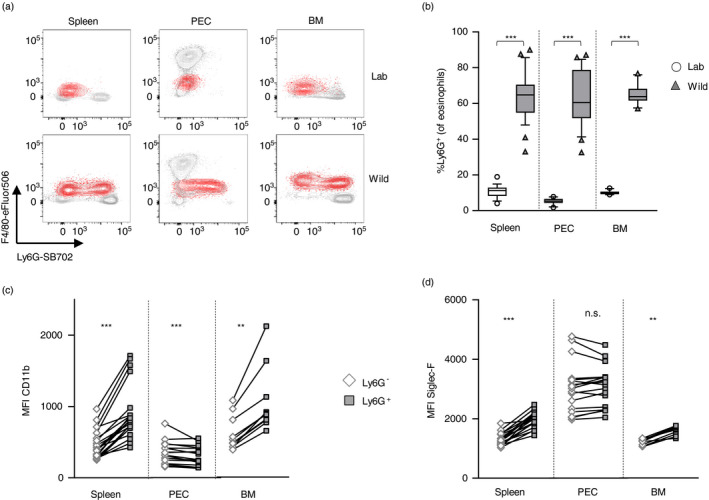
Majority of eosinophils in wild house mice express high levels of Ly6G. Spleens, peritoneal exudate cells (PEC) and bone marrow (BM) were collected from wild house mice from the Isle of May between September and December 2019, as well as from naïve C57BL/6 mice. (a) Example flow cytometric plots of F4/80 and Ly6G expression patterns of eosinophils (red) compared with other myeloid cell populations (grey). (b) Proportion of eosinophils expressing Ly6G. Mean fluorescent intensity (MFI) of (c) CD11b and (d) Siglec‐F expression in Ly6G^−^ and Ly6G^+^ eosinophils of wild mice. Box plots show median and quartiles with 10–90 percentile and residuals as dots. Laboratory versus wild data were analysed using a two‐tailed Mann–Whitney *U*‐test; n(Lab) = 16 (Spleen, PEC), 10 (BM); n(Wild) = 25 (Spleen, PEC), 10 (BM). Paired Ly6G^−^ versus Ly6G^+^ eosinophil data were analysed using a two‐tailed Wilcoxon matched‐pairs signed rank test, and the Holm's sequential Bonferroni procedure was applied to correct for multiple comparisons for each organ; n(Spleen) = 20, n(PEC) = 19, n(BM) = 10

The strikingly high Ly6G expression on the majority of wild eosinophils (median Ly6G positivity: spleen = 69%, PEC = 66%, BM = 64%) (Figure 3a,b) explains, in part, their previous classification as a cell type unique to wild mice [13], given that eosinophils are classically defined as Ly6G^−/low^ in naïve laboratory mice [23]. In fact, it is deemed good practice in laboratory research to exclude Ly6G^+^ cells in flow cytometric analysis in order to arrive at a pure eosinophil population. However, a handful of laboratory studies have reported the presence or induction of significant Ly6G expression on eosinophils in specific microenvironments. In particular, a small proportion of BM eosinophils has been shown to express Ly6G in an IL‐5‐dependent manner [24], which was amplified to around 40% by a fungal allergen challenge. Our findings suggest that immune mediators akin to IL‐5 may be influencing the bone marrow in wild mice. Enhanced fungal exposure in the wild could itself be a driver for changes in the granulocyte compartment, considering that re‐wilding of laboratory mice has been shown to increase intestinal colonization with fungi, which in turn induced an enrichment in circulating neutrophils [25]. Furthermore, Gr‐1^+^ (i.e. Ly6G^+^ and/or Ly6C^+^) eosinophils were found in spleens of alum‐primed mice [26], with roles in B‐cell priming put forward. Variable expression of Gr‐1 has also been noted on intestinal eosinophils, in the absence of Ly6C expression [27]. Adding to this literature, around 10% of Ly6G^+^ eosinophils have been reported to emerge in the lungs of allergen‐challenged mice. Ly6G^+^ eosinophils in this context exhibited a distinct expression pattern, with several immune mediators enriched in Ly6G^+^ eosinophils that were undetectable in Ly6G^−^ eosinophils, including CXCL13, IL‐27 and IL‐13 [28]. Eosinophil subsets, presumably with inflammatory or homeostatic phenotypes, have been reported in other instances including in the lung [29] and the gut [30]. Inclusion of Ly6G staining in studies further investigating phenotype and function of these eosinophil subsets would be pertinent, especially as Ly6G^+^ eosinophils have been reported in both of these tissues [27, 28]. Likewise, the investigation of eosinophil abundance and phenotype at mucosal sites, such as the intestine and the lung, in free‐living animals would be of utmost interest, including markers reported to distinguish eosinophil subsets in these tissues in laboratory mice [28, 29, 30].

The emergence of Ly6G^+^ eosinophils in laboratory mice, which have received an acute Th2‐type challenge, that is allergenic challenges or alum‐priming, led us to hypothesize that the distinctive Ly6G^+^ phenotype seen in wild eosinophils might be a marker of an enhanced activation state. We found that splenic and bone marrow eosinophils positive for Ly6G also showed increased expression levels of activation markers such as Siglec‐F and CD11b compared with Ly6G^−^ eosinophils (Figure 3c,d). However, in the peritoneal cavity, Ly6G expression did not associate positively with activation markers. CD11c expression on the other hand, another marker associated with activated eosinophils [31], was only positively associated with Ly6G expression in the peritoneal cavity (data not shown). Thus, markers that have been associated with eosinophil activation are not uniformly upregulated in Ly6G^+^ eosinophils, making Ly6G unlikely an indicator of activation *per se*. Alternatively, the presence of a high proportion of Ly6G^+^ eosinophils in the spleen, PEC and BM of wild mice may represent the early release of immature eosinophils from the bone marrow into the periphery. Ly6G is expressed on eosinophil progenitors during their maturation in the bone marrow [32], as well as on a recently described neutrophil precursor population with the plasticity to develop into eosinophils, at least *in vitro* [33]. Eosinophil progenitors are able to undergo maturation *in situ* following allergenic challenge and may therefore be seen in an immature state in the periphery [34]. The inclusion of immaturity markers such as CCR3 [35] in future studies may help answer this question.

**FIGURE 4:**
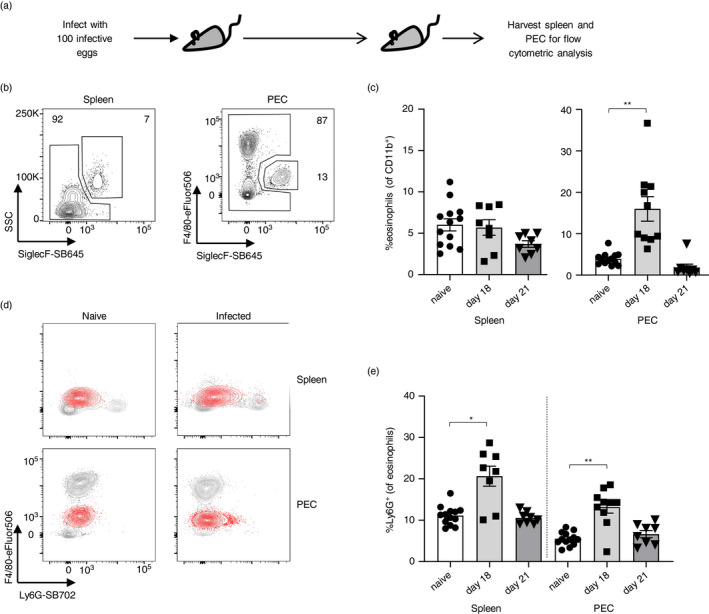
Eosinophils upregulate Ly6G expression during a high‐dose *T*. *muris* infection. (a) C57BL/6 mice were infected with 100 infective *Trichuris muris* eggs on day 0 or left untreated, and spleens and PECs were harvested on day 18 and d21, respectively. (b) Gating and (c) proportion of Siglec‐F^+^ eosinophils within myeloid cells. (d) Representative flow cytometric plots and (e) proportion of eosinophils expressing Ly6G. Data shown as mean +/− SEM. Naïve versus infected data were analysed using a Kruskal–Wallis test with Dunn's multiple comparisons test; *n* = 8–13 per group, pooled from 2 to 3 experiments

The Isle of May mice harbour a number of chronic parasitic infections including low levels of infection with the helminth *Trichuris muris* [19]. Helminth parasites are well known for their immunomodulatory capacity, although whether they affect eosinophil phenotype and function is unclear [36]. We therefore considered infections to be a potential driver for the observed Ly6G^+^ phenotype. To test whether a parasitic infection is able to drive eosinophil Ly6G expression, we infected laboratory C57BL/6 mice with a low dose of *T. muris* (30 infective eggs), thus mimicking infection intensities seen on the Isle of May. Low‐dose *T. muris* infections induce a Th1 response and lead to chronic infection [37]. However, neither during the larval stage of development (day 21) nor once adults were established (day 34) did the infection cause a significant change in eosinophil Ly6G expression (Figure S2). It is noteworthy that in a laboratory context, eosinophilia following *T. muris* infection is only prominent in high‐dose (100–200 eggs) acute infections [38]. In C57BL/6 mice, a high‐dose infection elicits a mixed Th1/Th2 response concomitant with transient eosinophilia and leads to parasite expulsion. Thus, as expected, during high‐dose infection (Figure 4a), there was a transient increase in eosinophil proportions in the PEC (Figure 4b) and notably a concomitant transient increase in the proportion of eosinophils expressing Ly6G in both spleen and PEC (Figure 4c,d). However, neither the proportion of eosinophils nor the expression levels of Ly6G were as enhanced as in wild mice, suggesting that there are stronger or multiple drivers of eosinophil Ly6G expression. For example, a genetic component to Ly6G expression on eosinophils has recently been revealed using an eosinophil‐specific deletion of the tribbles pseudokinase *Trib1*. *Trib1* is important in eosinophil identity and suppression of neutrophilic characteristics during development as well as in the mature eosinophil [39]. Whether the main driver for Ly6G expression on wild mouse eosinophils, both in our own wild mouse population and the mouse populations studied by Abolins et al. [13], is their genetic make‐up, other host‐intrinsic factors, host‐extrinsic factors or a combination thereof remains to be determined. However, the accumulating reports of the induction of Ly6G expression in laboratory mouse eosinophils [24, 27, 28], albeit at lower proportions than seen in wild mice, suggest that various environmental stimuli lead to the acquisition of this surface protein on eosinophils.

In conclusion, we describe for the first time the phenotype of eosinophils in a wild mouse population, which is enriched in immunologically relevant tissues. A large subset of these eosinophils exhibit a distinct phenotype marked by high Ly6G expression (Table S1), a feature rarely described in laboratory literature and its functional implication poorly understood. The function of Ly6G – even on the classical Ly6G^hi^ neutrophils – is unknown [40] and therefore warrants further investigation. Whilst parasitic infections may contribute to Ly6G expression by eosinophils, it is likely not the only driver. Our findings support the growing evidence that laboratory animals under current housing conditions do not accurately reflect the immune state of free‐living or ‘dirty’ animals, including humans [17, 41, 42]. Studying wild animals (in particular wild house mice) enables the capturing of naturally occurring immune phenotypes, which can guide our efforts to understand immune cell function in a complex, multifactorial environment. This could be a particularly fruitful avenue for the study of the eosinophil due to its involvement in highly context‐dependent processes such as tissue homeostasis and repair.

## CONFLICT OF INTEREST

The authors declare no competing financial interests.

## AUTHOR CONTRIBUTIONS

JEB and KJE conceived the overarching project. IM and KJE designed the current study and laboratory experiments. AW and JEB designed the fieldwork. AW, AL and IM organized the fieldwork. AW, IM, AL, JEB, KJE, AB, AM and JF collected field data. IM, HS and AM performed laboratory experiments. IM analysed the data. IM, KJE, AW and JEB interpreted the data. IM led the writing of the paper. IM and AW contributed equally to this publication and should be considered joint first authors. All authors contributed critically to the drafts and gave final approval for publication.

## ETHICAL APPROVAL

The work on wild mice was approved by the University of Nottingham Animal Welfare and Ethical Review Body and complies with the UK's Animals (Scientific Procedures) Act of 1986. The work on laboratory mice was approved by the University of Manchester Local Animal Welfare and Ethical Review Body and complies with the UK's Animals (Scientific Procedures) Act of 1986.

## Supporting information

Appendix S1
