# Supplementary material for: A lesson from the wild: The natural state of eosinophils is Ly6Ghi
Source: Immunology. 2021 Sep 15;164(4):766–76. doi: 10.1111/imm.13413 (PMC8561109; doi:10.1111/imm.13413)
Supplement: Supplementary file 1 — Appendix S1 [file IMM-164-766-s001.pdf]

Supplementary Figure 1

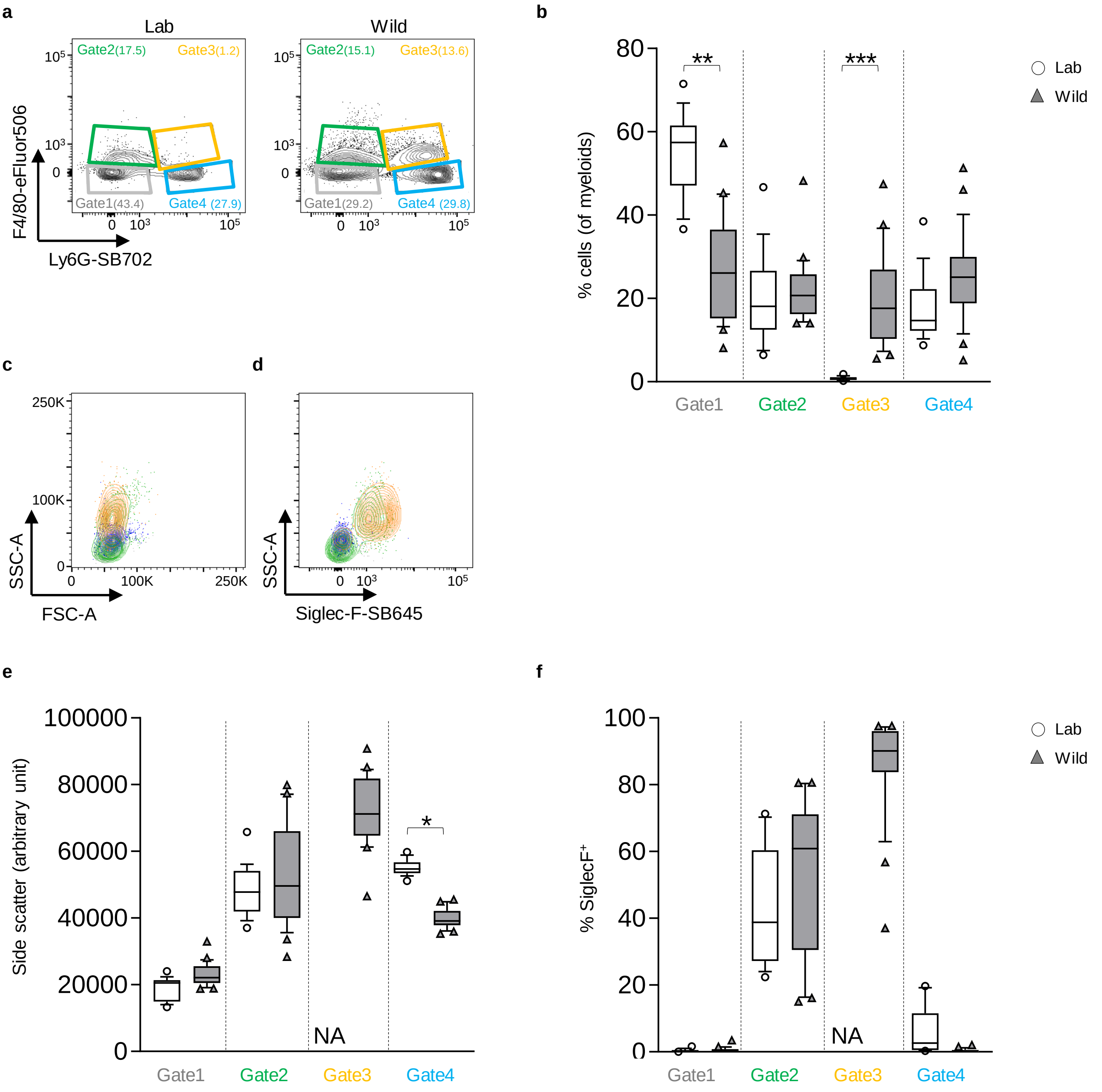

Supplementary Figure 1: F4/80⁺ Ly6G⁺ myeloid cells in wild mouse spleens are SiglecF⁺ and side-scatter^{hi}. Spleens were collected from wild house mice from the Isle of May between September and December 2019, as well as from naïve C57BL/6 mice. **(A)** F4/80 and Ly6G differentiates four myeloid populations in wild mice (1, grey; 2, green; 3, yellow; 4, blue). **(B)** Proportion of cells in gate 3 as defined in (A). **(C)** Side scatter and forward scatter characteristics of each myeloid population as defined in (A, Wild). **(D)** Siglec-F expression of each myeloid population as defined in (A, Wild). **(E)** Mean of side scatter in each myeloid population as defined in (A). **(F)** Proportion of Siglec-F^{+} cells in each myeloid population as defined in (A). Box plots show median and quartiles with 10-90 percentile and residuals as dots. Lab versus wild data analysed using a Kruskal-Wallis test with Dunn's post hoc test to correct for multiple comparisons; n(Lab) = 16, n(Wild) = 25.

Supplementary Figure 2

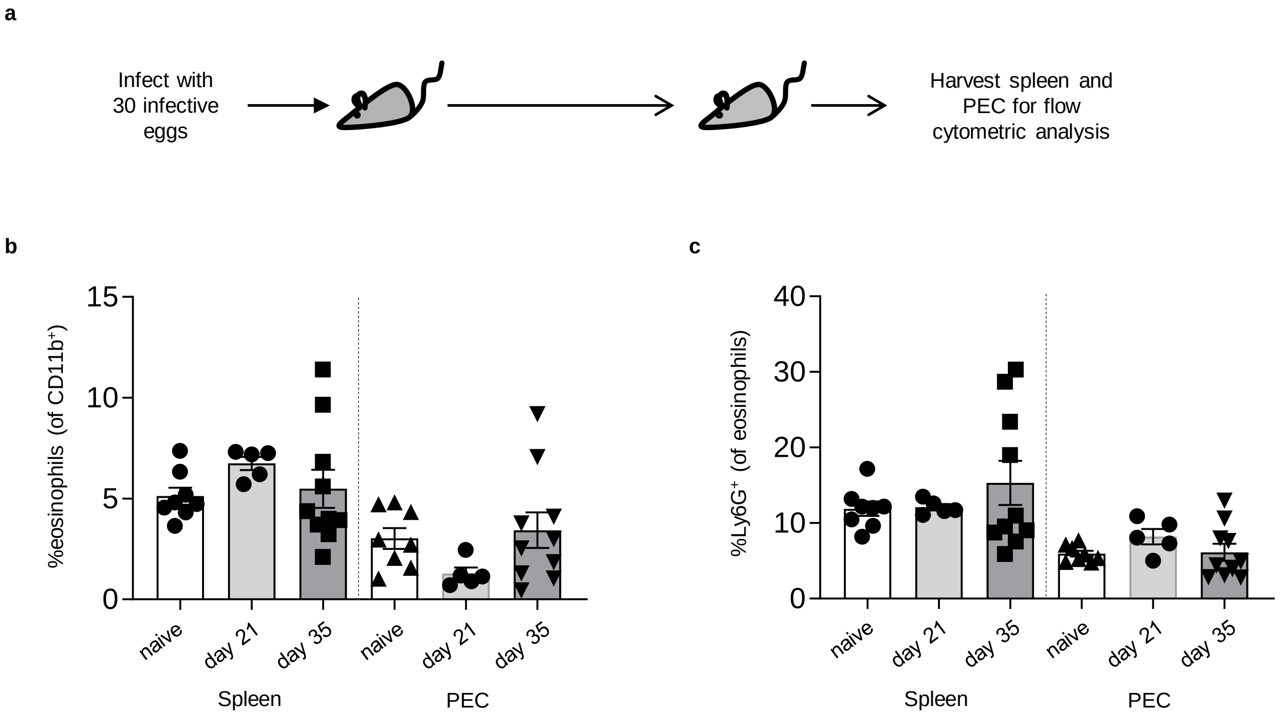

Supplementary Figure 2: Eosinophils in lab mice do not upregulate Ly6G expression during a low dose *T. muris* infection

(A) C57BL/6 mice were infected with 30 infective *Trichuris muris* eggs on day 0 or left untreated, and spleens and PECs harvested on day 21 or 35. **(B)** Proportion of SiglecF$^+$ eosinophils within myeloid cells. **(C)** Proportion of eosinophils expressing Ly6G. Data shown as mean +/- SEM. Naïve versus infected data was analysed using a Kruskal-Wallis test with Dunn’s multiple comparisons test; n = 5-10 per group, pooled from two experiments.

Supplementary Figure 3

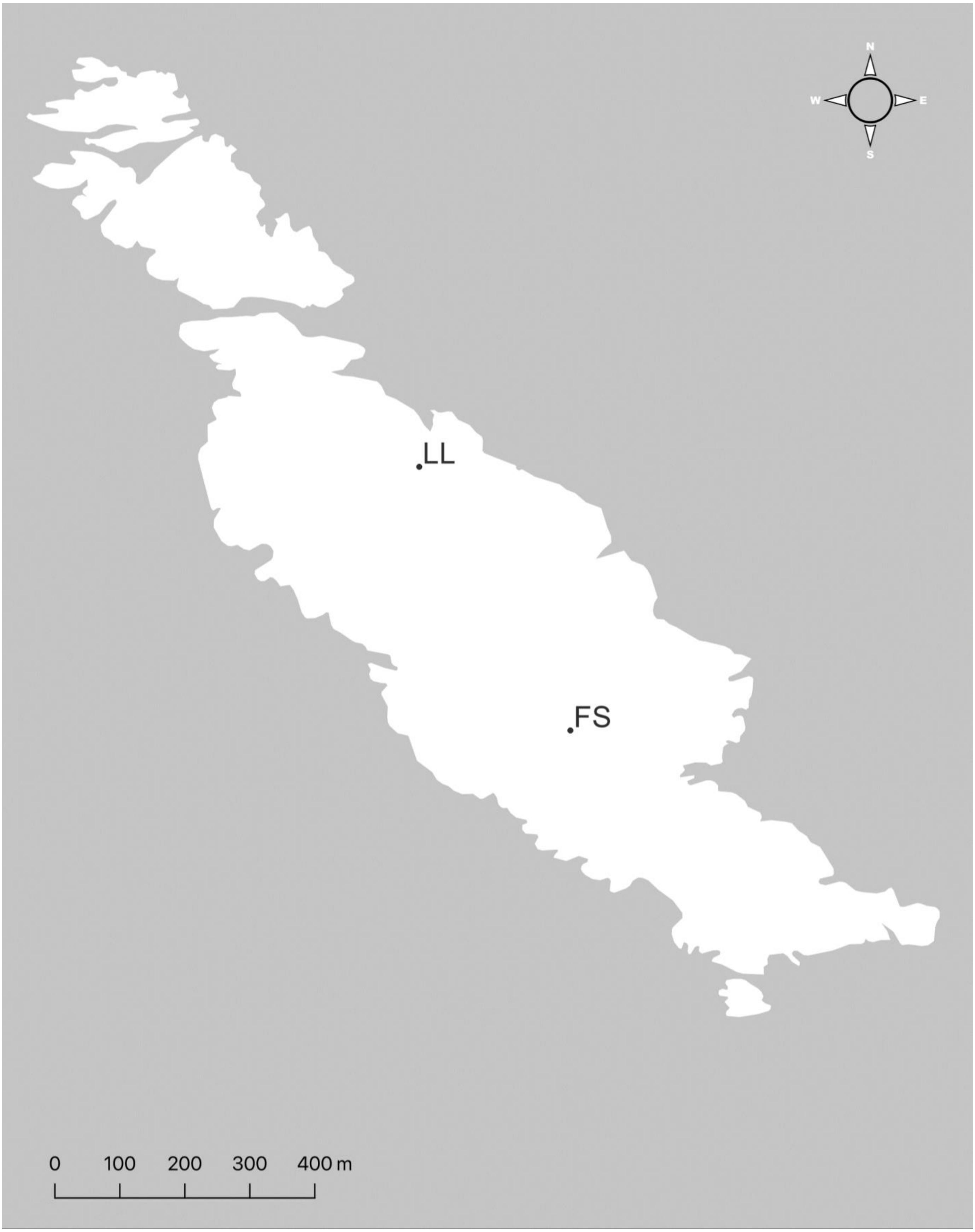

Supplementary Figure 3: Map of the Isle of May including the approximate locations of trapping sites. The Isle of May is located 8 km off the coast in the mouth of the Firth of Fourth, Scotland, UK (56°11'11.6"N, 2°33'24.1"W). It is about 1.8 km long and 0.5 km wide, covering an are of 45 ha. Both trapping sites, Fluke street (FS) and Low Light (LL), contained 6 parallel rows of 16 Longworth traps (96 traps in each grid), with traps 8-10 m apart.

Supplementary Figure 4

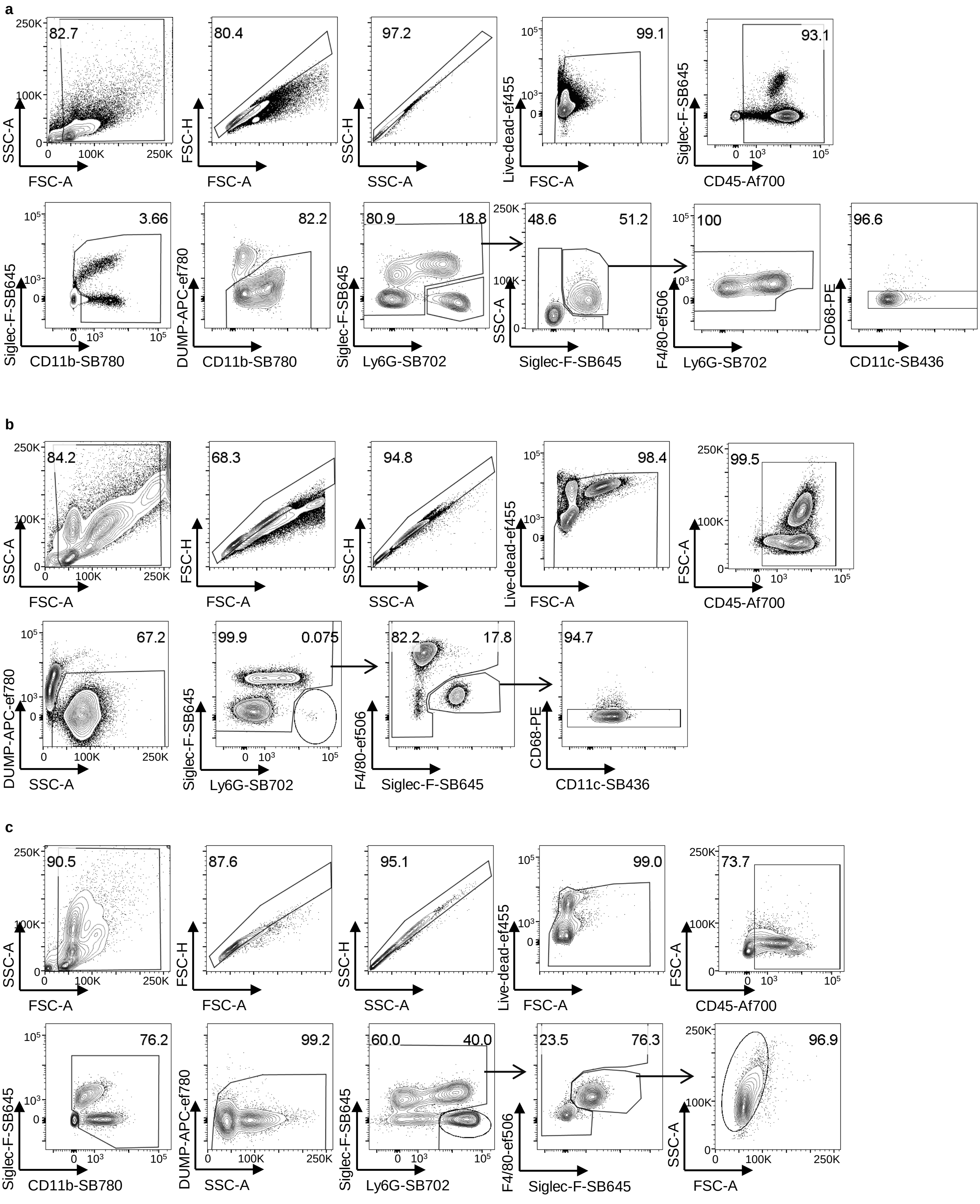

Supplementary Figure 4: Flow cytometric gating strategy for eosinophils in wild mice.

Spleens, peritoneal exudate cells (PEC) and bone marrow (BM) were collected from wild house mice from the Isle of May between September and December 2019 and stained for flow cytometric analysis. Gating strategy to obtain clean eosinophil population from **(A)** splenocytes, **(B)** PEC, and **(C)** BM of wild mice is displayed. For each organ, back-gating analysis and overlays were used to ensure that main myeloid cell populations, namely neutrophils, eosinophils, monocytes/macrophages and dendritic cells, were accounted for correctly throughout the gating strategy. DUMP channel included lineage markers CD3, CD19, and NKp46.

Supplementary Table 1

Phenotypic profile of eosinophils from spleen, PEC and BM of naïve lab mice and wild mice.

	Spleen		PEC		BM	
**Marker**	**Naïve lab**	**Wild**	**Naïve lab**	**Wild**	**Naïve lab**	**Wild**
Siglec-F	int/hi	int/hi	int/hi	int/hi	int/hi	int/hi
CD11b	lo/hi	lo/hi	lo/hi	lo/hi	lo/hi	lo/hi
F4/80	int	int	int	int	int	int
Ly6G	neg/lo	neg/pos	neg/lo	neg/pos	neg/lo	neg/pos
Ly6C	neg/pos	neg/pos	neg/pos	neg/pos	neg/pos	neg/pos
MHC-II	neg	neg	neg	neg	neg/lo	neg/lo
CD11c	neg/lo	neg/pos	neg/lo	neg/pos	neg/lo	neg/pos

Legend

Expression levels of surface antigens on eosinophils are classified as follows: neg = negative; lo = low; int = intermediate; hi = high; pos = positive**.** Dark shaded areas highlight major differences between naïve lab and wild mouse eosinophils. PEC = peritoneal exudate cells; BM = bone marrow cells.
